# Microglia drive diurnal variation in susceptibility to inflammatory blood-brain barrier breakdown

**DOI:** 10.1172/jci.insight.180081

**Published:** 2024-11-08

**Authors:** Jennifer H. Lawrence, Asha Patel, Melvin W. King, Collin J. Nadarajah, Richard Daneman, Erik S. Musiek

**Affiliations:** 1 Department of Neurology, Washington University School of Medicine, St. Louis, Missouri, USA.; 2 Department of Pharmacology, UCSD, San Diego, California, USA.; 3 Center On Biological Rhythms And Sleep (COBRAS), Washington University School of Medicine, St. Louis, Missouri, USA.

**Keywords:** Inflammation, Neuroscience, Innate immunity, Neurodegeneration, Neurological disorders

## Abstract

The blood-brain barrier (BBB) is critical for maintaining brain homeostasis but is susceptible to inflammatory dysfunction. While transporter-dependent efflux of some lipophilic substrates across the BBB shows circadian variation due to rhythmic transporter expression, basal transporter–independent permeability and leakage is nonrhythmic. Whether daily timing influences BBB permeability in response to inflammation is unknown. Here, we induced systemic inflammation through repeated LPS injections either in the morning (ZT1) or evening (ZT13) under standard lighting conditions; we then examined BBB permeability to a polar molecule that is not a transporter substrate, sodium fluorescein. We observed clear diurnal variation in inflammatory BBB permeability, with a striking increase in paracellular leak across the BBB specifically following evening LPS injection. Evening LPS led to persisting glia activation as well as inflammation in the brain that was not observed in the periphery. The exaggerated evening neuroinflammation and BBB disruption were suppressed by microglial depletion or through keeping mice in constant darkness. Our data show that diurnal rhythms in microglial inflammatory responses to LPS drive daily variability in BBB breakdown and reveal time of day as a key regulator of inflammatory BBB disruption.

## Abstract 2

Time-of-day influences microglial inflammatory responses and blood-brain barrier dysfunction in mice, with persisting glial activation and permeability observed with evening inflamogen exposure.

## Introduction

Activation of the innate immune response in the brain drives neuroinflammation, which is a critical component of many neurological disorders. Innate immune activation strongly influences many aspects of disease pathogenesis in age-related neurodegenerative disorders, including Alzheimer’s disease, as well as in more acute insults such as infection or stroke (1–4). In mice, inflammatory responses to a variety of inflammogens, including the TLR4 agonist LPS, show striking time-of-day (TOD) effects (5–8). Mice treated with systemic LPS in the evening have increased peripheral inflammation and mortality compared with those treated in the morning (5, 9, 10). The circadian clock system plays a role in regulating innate immune responses (11), and this TOD difference in LPS-mediated mortality can be ablated by disrupting the core circadian clock via deletion of the key clock gene *Bmal1* (5). However, LPS-induced inflammatory responses in the periphery, as well as animal mortality, are governed not only by the circadian clock but also a host of other factors including exposure to light and time of feeding, and they do not seem to rely on the circadian function of monocytes (5, 6, 11, 12). Moreover, while diurnal variation in the severity of innate immune activation has been demonstrated in peripheral organs, it is unknown if similar TOD variations in the severity of inflammation occur in the brain and, if so, which cells might regulate this phenomenon.

One consequence of neuroinflammation is dysfunction of the blood-brain barrier (BBB). The BBB is critical for restricting certain blood-borne molecules from the brain, helping protect the CNS from circulating neurotoxic and neuroinflammatory molecules as well as peripheral immune cells (13–15). The BBB is a multicellular structure, consisting of endothelial cells, mural cells (pericytes and smooth muscle cells), and astrocytes — collectively referred to as the neurovascular unit (NVU). Outside of the NVU, microglia and neurons also affect BBB function (16, 17). This selectively permeable barrier is formed by a series of distinct cellular properties within the NVU, including continuous nonfenestrated vessels, decreased pinocytosis, the presence of junctional proteins, and additional regulatory elements that allow for the selective movement of solutes between the blood and CNS (18). In the setting of peripheral or central inflammation, the BBB can become more permeable due to transporter dysfunction, breakdown of endothelial cell tight and adherens junctions, and increases in transcytosis (19, 20). BBB permeability to lipophilic molecules that are substrates for specific transporter pumps has been reported and is dependent on circadian expression of those transporter proteins, such as p-glycoprotein (17, 21). However, the BBB does not show daily rhythms in permeability or leakage of nonsubstrate molecules under basal conditions (17, 22–24). It is unknown how disease processes such as inflammation might influence TOD variations in BBB permeability, as many cellular functions can gain diurnal rhythms in the setting of pathology (25–28).

Here, we have investigated how neuroinflammatory response and BBB permeability to polar, nontransporter substrate molecules (termed inflammatory BBB breakdown) are influenced by innate immune activation (exposure to i.p. LPS injection) at different TODs. We observed that both inflammatory gene expression in the brain and BBB breakdown are both much more severe following LPS exposure in the evening (zeitgeber time 13 [ZT13]) as compared with morning (ZT1). This diurnal variation appears to depend on light exposure and is characterized by persisting astrocyte and microglial activation and cytokine production, which are not observed in the morning and is not present in peripheral organs. We find that exaggerated neuroinflammatory BBB disruption in the evening occurs independently of rhythms in peripheral inflammation and is instead intrinsic to CNS-resident immune cells, particularly microglia.

## Results

### TOD strongly regulates inflammatory BBB breakdown.

We first sought to determine if exposure to an inflammatory stimulus at different TODs can differentially affect BBB permeability. Based on published data showing increased mortality to i.p. injection of LPS given in the evening, we used an evening time of injection (ZT13, or 1 hour after lights-off) to pilot different LPS dosing regimens on BBB permeability to sodium fluorescein (NaFL), a commonly used tracer for BBB permeability studies that is polar and has no known endothelial transcellular transport proteins (29). We observed that a single i.p. 3 mg/kg LPS dose at ZT13 caused minimal increase in BBB permeability, while a 5 mg/kg dose induced some BBB permeability but also mortality. Thus, we tried a lower dose (2 mg/kg) given on 2 consecutive days (at ZT13) and found that this dosing scheme elicited consistent increases in BBB permeability (Supplemental Figure 1; supplemental material available online with this article; https://doi.org/10.1172/jci.insight.180081DS1) with negligible mortality. Therefore we used the 2 mg/kg i.p. dose (LPS or PBS control) given on 2 consecutive days at the same TOD, with BBB permeability measurement 24 hours after the second dose, throughout the study.

**Figure 1:**
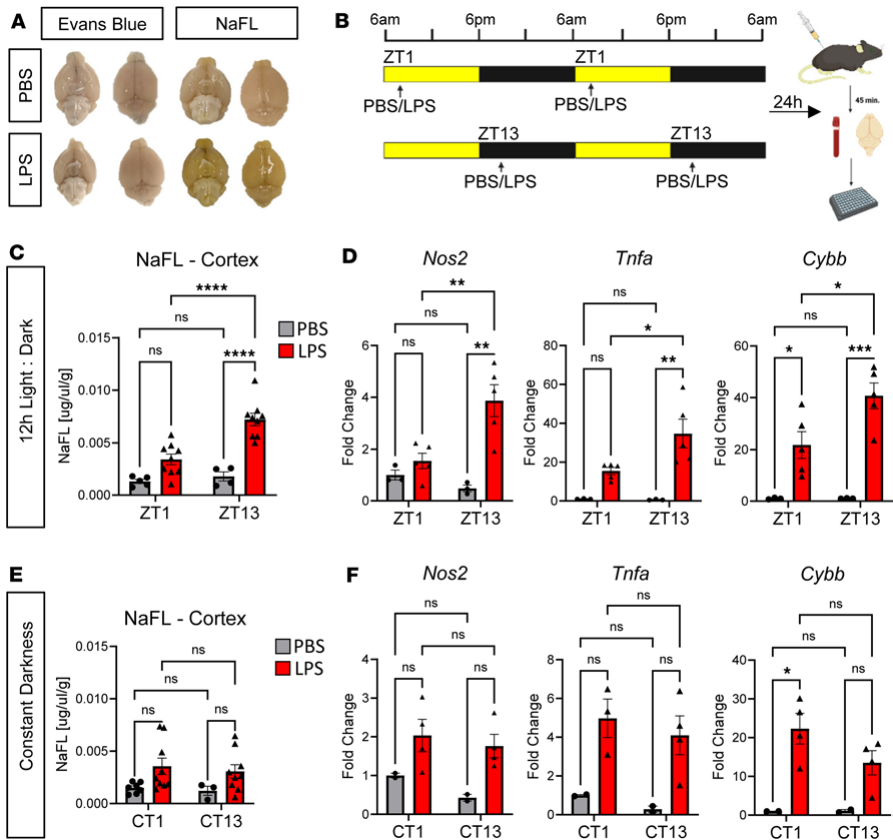
Diurnal variation in inflammatory BBB breakdown. (**A**) Representative images of dorsal and ventral view of whole brains from mice injected with PBS and LPS before being given either EB or sodium fluorescein (NaFL) tracer. (**B**) Schematic of the 2-hit LPS experimental paradigm used for diurnal BBB permeability experiments using NaFL, with lights-on (ZT0) occurring at 6 a.m. and lights-off (ZT12) occurring at 6 p.m. (**C**) Quantification of diurnal differences in LPS-induced NaFL leak in the cortex for mice kept in 12h Light:Dark (L:D). For L:D data, both main effects of treatment and time of day, as well as interaction, were significant by 2-way ANOVA. Post hoc test *P* values are shown. (**D**) qPCR data from cortex of PBS or LPS treated mice under L:D conditions. *n* = 3–5 mice per group. Main effect of treatment and interaction were significant by 2-way ANOVA; post hoc *P* values are shown. (**E**) Quantification of diurnal differences in LPS-induced NaFL leak in the cortex for mice kept in D:D. No main effect was significant by 2-way ANOVA. (**F**) qPCR data from cortex of PBS or LPS-treated mice under D:D conditions. *n* = 2–5 mice per group. For all panels, each circle is *n* = 1 mouse. *P < 0.05, **P < 0.01, ***P < 0.005, ****P < 0.001.

In order to identify gross size exclusion properties in LPS-induced BBB breakdown, we examined BBB permeability to a second polar tracer, Evans blue (EB), in mice treated exclusively at ZT13, as above. EB is a relatively small azo dye (961 Da) that binds with high affinity to serum albumin, creating a high molecular weight (mw) tracer of 69 kDa (30). Conversely, NaFL is a small mw (376 Da) inert fluorescent molecule that weakly binds to endogenous proteins and thus is much smaller (29). Neither phosphate buffer saline (PBS) control nor LPS-treated mice showed any visible leak of EB, indicating there is no gross hemorrhaging or permeability to larger molecules in this model of inflammation (Figure 1A). Of note, we observed no increase in permeability to larger dextran molecules (3 kDa or 70 kDa; data not shown) hpi, while biotin (0.2 kDa) appeared to label blood vessels more readily in LPS-treated mice (Supplemental Figure 1). We chose NaFL for subsequent experiments because it showed increased permeability after i.p. LPS and was more inert than biotin.

To examine the effect of TOD on inflammatory BBB breakdown, we administered 2 mg/kg LPS or PBS control to 2-month-old male C57BL/6 mice for 2 days in a row (i.e., “2-hit model”) at either zeigeber time (ZT) 1 (7 a.m.) or ZT13 (7 p.m.) (Figure 1B). In a 12-hour light/12-hour dark cycle with the lights on at 6 a.m., these times correspond to 1 hour after the onset of light (ZT1) and 1 hour after the lights turn off (ZT13). We chose these times because they roughly correspond to the nadir and peak of diurnal behavioral rhythms, as well as circadian gene expression in most organs (31). To measure BBB breakdown, we administered s.c. NaFL on the third day at the same TOD as prior LPS injections and quantified the amount of tracer found in the hippocampus, cortex, and cerebellum, normalized to the amount found in the serum (Figure 1B). As expected, there was minimal permeability of NaFL in PBS-treated mice at either ZT1 or ZT13 (Figure 1C), supporting previous studies stating that basal permeability to polar molecules that are not transporter substrates does not show diurnal variation (17, 22). However, mice treated with LPS in the evening (ZT13) had significantly greater NaFL leak across multiple brain regions compared with those treated in the morning (ZT1), with the cerebellum having the greatest absolute leak and the cortex having the strongest TOD effect (Figure 1C and Supplemental Figure 2A). Of note, while LPS treatment at ZT1 only slightly increased BBB permeability to NaFL in the cortex and hippocampus, permeability was significantly increased in all brain regions hpi exposure at ZT13. We examined expression of transcripts associated with inflammation (*Nos2*, *Tnfa*) and microglial activation (*Cybb*) by quantitative PCR (qPCR) in cortex tissue taken from mice at the same time as the BBB permeability assay and found that LPS induced these 3 genes to a significantly greater degree in the ZT13 group, suggesting that evening LPS exposure is associated with both elevated neuroinflammation and BBB breakdown (Figure 1D). Indeed, *Nos2* and *Tnfa* increases were exclusively seen in the ZT13 group.

Since constant darkness is known to abrogate the TOD effect of LPS-induced mortality (5), we next examined the effects of 24 hours of constant darkness (D:D) on LPS-induced BBB breakdown. We found that the diurnal differences in LPS-induced NaFL leak were ablated in all 3 brain regions in mice kept in D:D for 24 hours prior to the first injection and until perfusion (Figure 1E and Supplemental Figure 2B), suggesting that light/dark conditions are required for diurnal increases in LPS-induced inflammatory BBB breakdown. We also found that D:D ablated TOD difference in LPS-induced *Nos2*, *Tnfa*, and *Cybb* gene expression, suggesting a general reduction of neuroinflammation (Figure 1F). Altogether, these data indicate that light/dark conditions are required for optimal diurnal variation in neuroinflammation and inflammatory BBB breakdown.

While inflammatory BBB breakdown and circadian rhythms in barrier properties have both been explored independently, there is relatively little known about daily rhythms in neuroinflammation and BBB breakdown. Our current findings suggest that there are diurnal changes in the susceptibility of the BBB to inflammatory breakdown, beyond the known rhythms in transporter activity (17, 22–24). Previous studies on LPS-induced mortality have found that the phenomenon is light dependent and myeloid cell clock independent, as rhythms in mortality are ablated in mice kept in D:D and persist in mice deficient in the master circadian clock protein, BMAL1, in myeloid cells (5). Similarly, we found mice kept in D:D showed no diurnal variation in BBB breakdown or in markers of neuroinflammation or microgliosis. Of note, constitutive brain-specific deletion of BMAL1 has been reported to induce BBB breakdown at baseline, further complicating the study of cellular circadian rhythms in inflammatory BBB leakage (32).

### Evening LPS exposure disrupts endothelial cell morphology.

**Figure 2:**
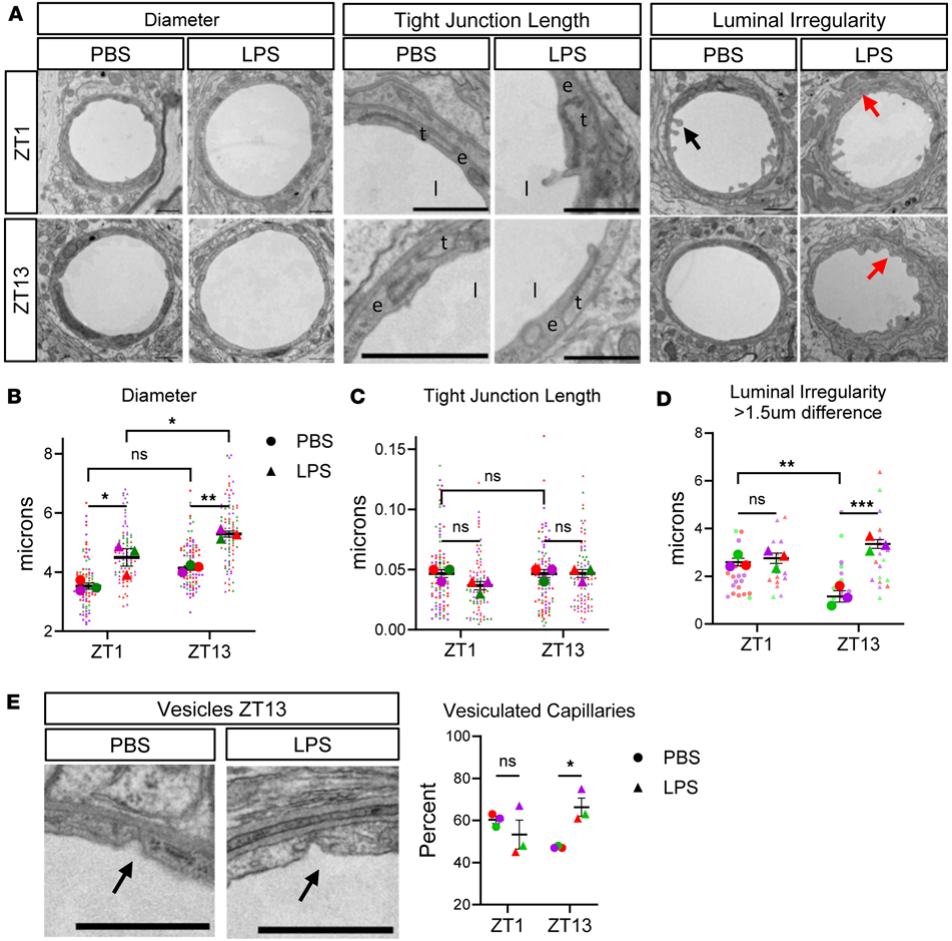
Evening LPS exposure disrupts endothelial cell morphology. (**A**) Representative images of diameter, tight junction length (e, endothelial cell; l, lumen; t, tight junction), and luminal irregularity (arrows: black showing blebbing at luminal surface, red showing plasma/basement membrane disruption). (**B**) Quantification of diameter length across cortical capillaries; main effect of LPS was significant, but interaction was not, by 2-way ANOVA. (**C**) Quantification of average tight junction length per capillary; no effects were significant by 2-way ANOVA. (**D**) Quantification of luminal irregularity; main effect of TOD and interaction were significant by 2-way ANOVA and post hoc test. (**E**) Inclusion criteria for vesicles (arrow indicating invaginating vesicles) and quantification of percentage of capillaries that contain 1 or more vesicles. Interaction was significant by 2-way ANOVA and post hoc test. For all graphs, *n* = 3 mice per group, 28–32 capillaries per mouse. Average for each mouse is shown in large dots, while technical replicates shown as smaller dots in similar color per mouse. Scale bar: 1 μm.

We next used transmission electron microscopy (TEM) to image cortical capillary ultrastructure (described in Supplemental Figure 2C). Solutes can cross the BBB in a myriad of ways including passive paracellular or transcellular diffusion as well as adsorptive-, carrier-, or receptor-mediated transport. Inflammatory BBB breakdown occurs primarily through either disruption of endothelial tight junctions (TJs) or an increase in transcytosis (33). While we saw no gross disruption of the NVU, there was significant diurnal variation in vasodilation, with an increase in capillary diameter in mice treated with LPS at ZT13 as compared with ZT1 (Figure 2, A and B), consistent with the increase in *Nos2* and *Vasp* seen in our RNA-Seq dataset. Despite known circadian rhythms in *Claudin5* gene expression (34), we measured the average TJ length per vessel and saw no change based on time or treatment (Figure 2, A and C). However, we did note select disrupted TJs imaged in the LPS treatment group (Supplemental Figure 2D).

Previous TEM studies have also found that LPS induces luminal plasma membrane ruffling, a mechanism of nonreceptor-mediated pinocytosis used by bacteria and viruses to increase infection of epithelial cells (20, 35, 36). We found a significant increase in severe luminal irregularity in LPS-treated mice at ZT13 only (Figure 2, A and D). To confirm that the increase in plasma membrane disruption seen in ZT13 LPS-treated mice is indicative of increased transcytosis, we next quantified the number of vesicles per capillary, only counting actively invaginating vesicles from the luminal side. We found a significant increase in the percentage of vessels containing at least 1 vesicle in LPS-treated mice at ZT13 only (Figure 2E). Altogether, these ultrastructural abnormalities indicate that evening LPS exposure induces increased vasodilation as well as a disrupted endothelial cell morphology that is consistent with increased paracellular or transcytosis-mediated particle uptake.

### Evening LPS exposure causes persisting neuroinflammation and gliosis.

**Figure 3:**
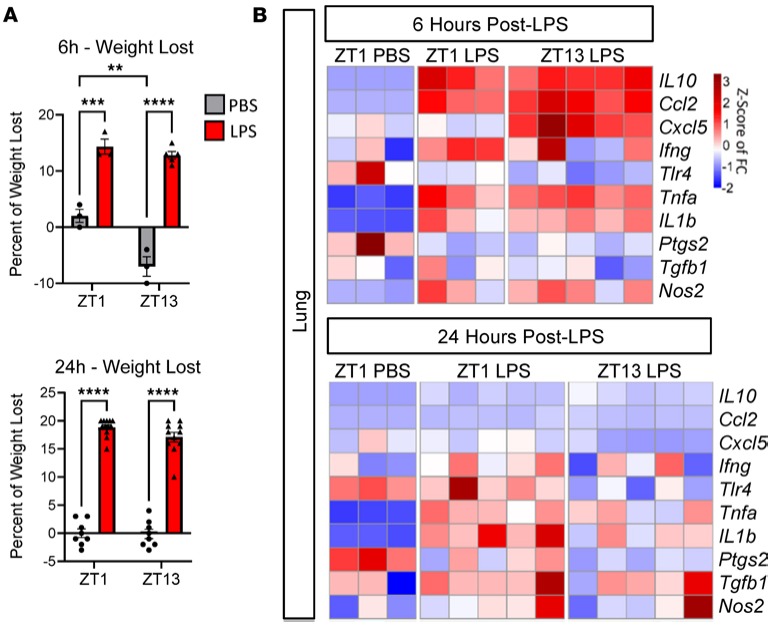
Absence of diurnal variation in peripheral immune activation at 6 or 24 hpi. (**A**) Percent weight lost in mice treated with PBS or LPS at ZT1 or ZT13, measured either 6 or 24 hpi. (**B**) Heatmap of selected gene expression in mouse lung tissue 6 versus 24 hours after final LPS injection at ZT1 or ZT12. Gene expression is normalized to ZT1 PBS 6 hpi group. **P < 0.01, ***P < 0.005, ****P < 0.001. In **A** and **B**, the main effect of LPS was significant by 2-way ANOVA. Post-hoc test results are shown.

We next sought to examine how exposure to an inflammatory stimulus at different TODs differentially affects CNS inflammation using the same 2-day ZT1/ZT13 i.p. LPS model. In models of systemic LPS administration, peripheral cytokine mRNA levels peak between 3 and 6 hours postinjection (hpi) (37–40), so we collected peripheral and CNS tissue 6 and 24 hours after the final ZT1 or ZT13 dose of LPS (6 hour time points at LPS injection were collected at 1 p.m. [ZT7] or 1 a.m. [ZT17]) and examined mRNA expression of a subset of inflammation-related transcripts by qPCR. We first examined the peripheral effects of LPS using lung tissue due to its known circadian rhythmicity in LPS response with minimal effect of time of feeding (6, 11). We saw no diurnal differences in percent weight loss between the ZT1- and ZT13-injected groups at either 6 or 24 hours hpi (Figure 3A). While some inflammatory transcripts were induced by LPS in lung, we observed no differences between the ZT1 and ZT13 groups, at either 6 or 24 hpi (Figure 3B). Additionally, we saw no diurnal differences in inflammatory gene expression in the liver at 6 hpi or the spleen at 24 hpi (Supplemental Figure 3); altogether, these data indicate that, in this particular “2-hit” model of systemic inflammation and at these time points, there is no evidence of major diurnal variations in peripheral inflammatory response.

**Figure 4:**
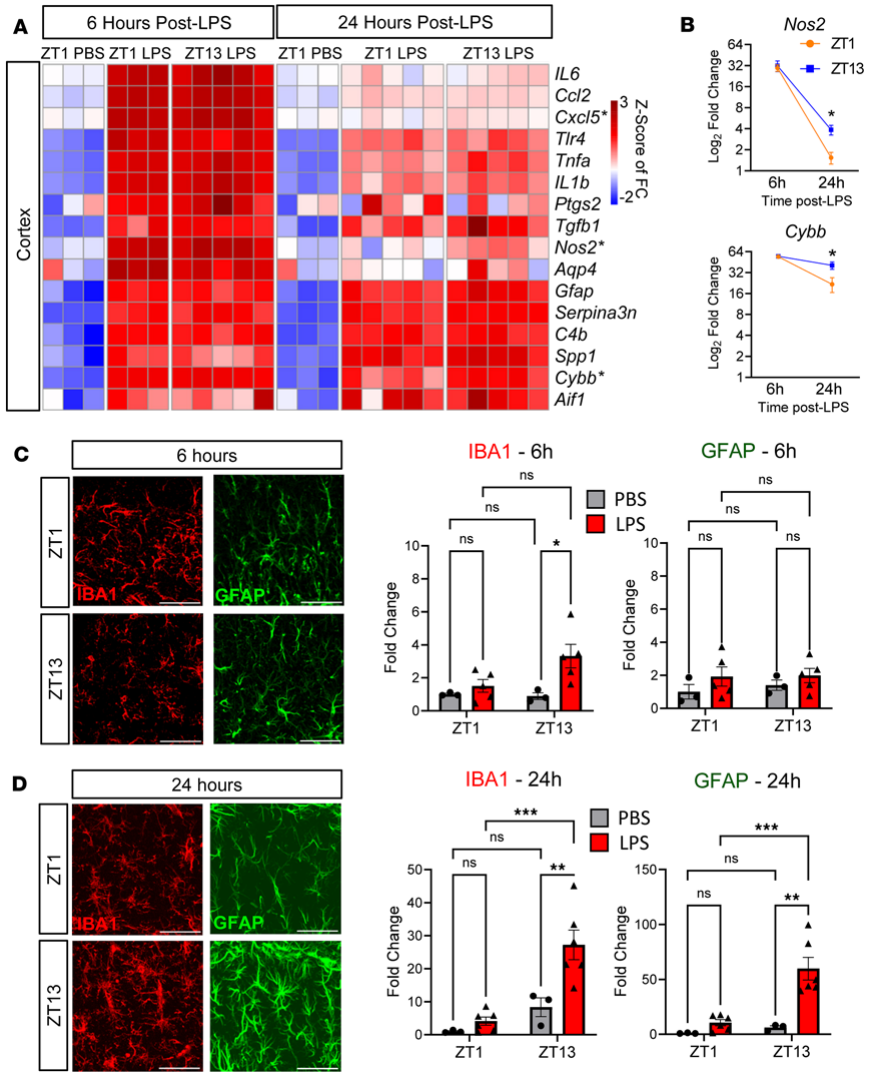
Evening LPS causes persisting inflammation and gliosis. (**A**) Heatmap of selected gene expression in mouse cortex 6 versus 24 hours after final LPS injection at ZT1 or ZT12. Gene expression is normalized to ZT1 PBS 6 hpi group. Transcripts with significant time-of-day (TOD) effect at 24 hpi are denoted with asterisks. Scale is *Z* score of Log_2_FC. (**B**) *Nos2* and *Cybb* graphed across time. (**C**) Representative images of IBA1 and GFAP after 6 hours of PBS or LPS treatment with quantification of percent area in the hippocampus 6 or 24 hpi, normalized to ZT1 PBS group. (**D**) Representative images of IBA1 and GFAP after 24 hours of PBS or LPS treatment with quantification of percent area in the hippocampus 6 or 24 hpi, normalized to ZT1 PBS group. *n* = 3–5 mice per group. For the 6-hour time point, only main effect of LPS was significant for IBA1, while in the 24-hour time point, the main effect of LPS and interaction are significant for both proteins; post hoc test results are shown. *P < 0.05, **P < 0.01, ***P < 0.005. Scale bars: 50 μm.

We next examined neuroinflammatory gene expression in the same mouse cohort. In cerebral cortex tissue, while LPS strongly induced inflammatory gene expression at 6 hpi, diurnal differences were not evident. At 24 hpi, overall expression of some inflammatory genes had waned, but several inflammatory genes (*Tnfa*, *Cxcl5*, and *Nos2*) showed increased expression in the Z13 LPS group, as compared with the ZT1 group (Figure 4A). When comparing the 6 hpi to 24 hpi gene expression datasets, there is a sharp reduction in cortical inflammation at 24 hpi in all groups, though this resolution of inflammatory gene expression is dampened in the ZT13 LPS group (Figure 4, A and B). Conversely, markers of gliosis remain persistently elevated at both 6 and 24 hpi, with the microglial activation marker *Cybb* gaining a diurnal difference 24 hpi (Figure 4, A and B). We next quantified micro- and astrogliosis by IHC in the same groups of mice (ZT1 or ZT3 LPS) sacrificed at 6 or 24 hpi. At 6 hpi, there were no diurnal effects on microgliosis as measured by IBA1 percent area in the hippocampus, although there was a treatment effect at only ZT13 (Figure 4C). Similarly, using GFAP as a marker for astrogliosis in the hippocampus, there were no LPS or time effects found at 6 hpi (Figure 4C). However, there was a significant diurnal effect on IBA1 and GFAP immunoreactivity 24 hpi, with both microglia and astrocytes showing increased activation in the ZT13 LPS group (Figure 4D). These data suggest that there may be decreased resolution of inflammation and gliosis in mice treated with LPS at ZT13 compared with those at ZT1.

### Persisting neuroinflammation after evening LPS specifically enhances BBB-related gene expression.

**Figure 5:**
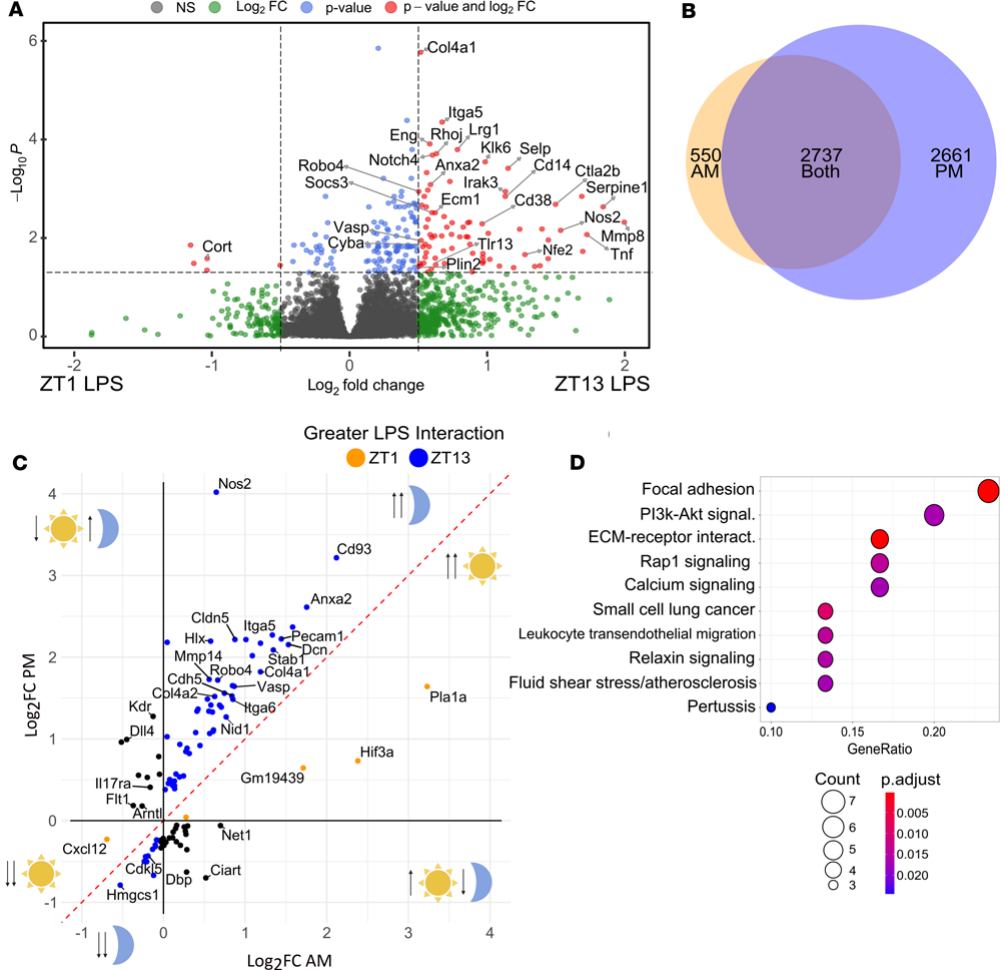
Persisting neuroinflammation after evening LPS exposure specifically enhances BBB-related gene expression. (**A**) Volcano plot of differentially expressed genes (DEGs) from bulk RNA-Seq in LPS-treated mouse cortex, comparing ZT1 versus ZT13 treatment time. Red dots indicate transcripts with adjusted *P* < 0.05 and fold change > 1.75-fold; right upper area indicates higher expression in mice treated at ZT13. (**B**) Venn diagram showing overlap of DEGs upregulated hpi in the ZT1 versus ZT13 datasets. (**C**) Scatter plot of genes with a significant TOD and treated interaction by 2-way ANOVA. Log fold change (PBS versus LPS) in the a.m. (ZT1) group is shown on *x* axis and in the p.m. (ZT13) group on *y* axis. Illustrations indicate the response of genes in that area to LPS (up or down arrow) in the a.m. (sun) or p.m. (moon). Genes shown above the red dotted line and right of the *y* axis were all upregulated in the p.m., with *Nos2* being the highest fold change. (**D**) ORA dotplot of the top dysregulated KEGG pathways using the full gene list from **D**. *n* = 3–5 mice per group.

To more fully characterize i.p. LPS-induced neuroinflammation 24 hpi, we conducted bulk RNA-Seq on cortex samples from mice treated with i.p. LPS (or PBS control) at ZT1 and ZT13. While previous studies have characterized the brain inflammatory profile induced by LPS, none have considered diurnal variations in immune response to LPS exposure (41–43). When comparing only LPS-treated mice between ZT1 and ZT13, we identified 209 differentially expressed genes (DEGs; 181 upregulated and 28 downregulated, adjusted *P* < 0.05) in the ZT13 LPS compared with ZT1 LPS group (Figure 5A). Many of these genes upregulated in the evening were proinflammatory mediators (*Nos2*, *Mmp8*, *Stab1*, *Cd14*). Since the majority of DEGs were upregulated at ZT13, we next asked if LPS treatment induced more transcripts at ZT13 than at ZT1 by examining PBS versus LPS at ZT1 and separately analyzing PBS versus LPS at ZT13, before comparing the DEG lists. There were 3,287 DEGs (1,761 upregulated and 1,526 downregulated) in LPS compared with PBS treated mice at ZT1 and 5,398 DEGs (2,756 upregulated and 2,642 downregulated) in LPS compared with PBS treated mice at ZT13. Comparisons between these 2 datasets found that there were 2,661 DEGs uniquely found in mice treated at ZT13, with only 550 DEGs unique to ZT1, and an additional 2,737 DEGs found at both times (Figure 5B).

We next used a 2-way ANOVA to examine statistical interaction between our 2 variables, TOD (ZT1 versus ZT13) versus treatment (PBS versus LPS). This method allowed us to control for baseline rhythms in gene expression in the PBS control groups. We then plotted only the DEGs with a significant TOD × LPS interaction on axes representing their log_2_fold change in the morning (*x* axis) versus evening (*y* axis). This showed us that DEGs that are upregulated in response to LPS tend to be further upregulated when treated with LPS in the p.m. (Figure 5C). KEGG pathway analysis of all DEGs with a significant TOD × treatment interaction found dysregulation of several vascular-related functions — focal adhesion and fluid shear stress and atherosclerosis — as well as inflammatory pathways, including leukocyte transendothelial migration and ECM-receptor interaction (Figure 5D). Interestingly, many of the genes with a TOD × treatment interaction are involved in BBB function, including upregulation of the TJ- and AJ-associated proteins *Cldn5* and *Cdh5* (Figure 5C). Additionally, there was a diurnal increase in expression of the *Vasp* and inducible *Nos2* genes, both of which have been implicated in BBB dynamics (Figure 5C) (44, 45). Altogether, while we saw significant changes in LPS-induced gene expression at both the ZT1 and ZT13 time points, BBB-associated genes were among the top dysregulated pathways when considering the interaction of time and treatment.

### Microglia are required for LPS-induced neuroinflammatory BBB breakdown at ZT13.

**Figure 6:**
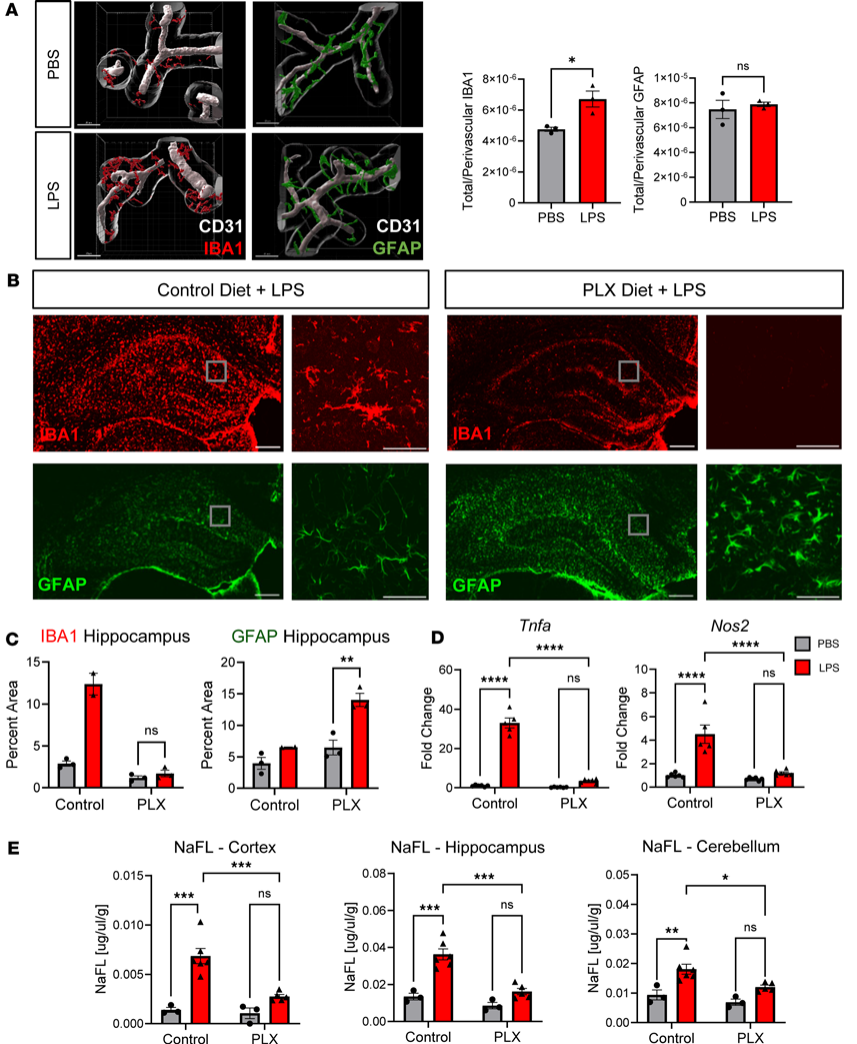
Microglia are required for LPS-induced inflammatory BBB breakdown at ZT13. (**A**) Volume of IBA1 and GFAP colocalized within 8 μm of CD31, normalized to total volume, at ZT13 24 hpi. *n* = 3 mice per group; 3–6 vessels imaged per mouse. (**B**) Representative images of hippocampal IBA1 and GFAP staining in control and PLX LPS-treated mice. Inset images are of the hippocampus at 40× magnification. (**C**) Quantification of IBA1 and GFAP hippocampal percent area. *n* = 2–3 mice per group. (**D**) LPS-induced cortical cytokine expression in PBS or LPS-treated mice given control or PLX chow. *n* = 5–6 mice per group. (**E**) NaFL assay in PBS or LPS-treated mice given either PLX or control chow. Scale bars: 200 μm (**B**), 50 μm (inset).

At baseline, capillary-associated microglia play an active role in maintaining BBB integrity. Ablating microglia (and other CSFR1^+^ myeloid populations in the brain) with the CSFR1 antagonist Plexidartinib (Plexidartinib 3397 [PLX3397, hereafter referred to as PLX]) alters vascular tone without changing pericyte coverage or astrocyte endfoot density (46, 47). During inflammation, PLX treatment protects against LPS-induced inner blood retinal barrier leak in the eye but is deleterious during models of both ischemic stroke and microbubble-induced hemorrhage in the CNS (16, 48, 49). Additionally, in our LPS model, we found that the increase in gliosis seen at ZT13 was coupled with increased perivascular localization (within 8 μm of a blood vessel) of IBA1 but not GFAP (Figure 6A). To determine if microglia are required for LPS-induced BBB breakdown, we treated mice for 2 weeks with PLX prior to LPS administration. Since there was no significant increase in BBB permeability with LPS treatment at ZT1 (Figure 1C and Supplemental Figure 2A), we only treated PLX-fed (or control chow–fed) mice at the ZT13 time point. Of note, a previous study shows that PLX administration does not affect behavioral circadian rhythms in mice (50). As previously reported, we found that PLX treatment dramatically reduced the number of IBA1^+^ cells (Figure 6, B and C) (47) in both PBS- and LPS-treated groups without altering peripheral immune responses or weight loss (Supplemental Figure 4A) (51). Despite microglia being the primary TLR4^+^ CNS-resident cell, LPS-treated PLX mice had increased hippocampal GFAP (Figure 6, B and C) as well as *Serapina3n* and *Gfap* upregulation (Supplemental Figure 4B), suggesting that astrocyte activation in response to systemic LPS is independent of microglial function. However, despite these changes in astrocyte GFAP expression, PLX administration reduced LPS-induced *Tnfa* and *Nos2* in the cerebral cortex and completely protected against NaFL leak, indicating that microglia are required for neuroinflammation following peripheral LPS injection, as well as inflammatory BBB breakdown (Figure 6, D and E).

We next sought to manipulate astrocyte activation to determine its role in neuroinflammation caused by evening LPS exposure. Since astrocytes are known to mitigate microglial reactivity after CNS injury and are in close proximity to blood vessels, they are prime candidates to serve as the first responders to LPS-induced peripheral cytokines (52). Activation of the JAK2/STAT3 pathway is a critical step in astrocyte activation in disease models (53). In vitro, LPS stimulation induces an astrocytic STAT3-dependent release of TNF-α, leading to a loss of endothelial cell barrier properties (54). Thus, we chose to target the astrocyte STAT3 pathway in our model, utilizing a previously described AAV9 viral vector that expresses a constitutively active form of JAK2 (JAK2ca) behind a truncated GFAP promoter (55). Activated JAK2 phosphorylates STAT3, which then translocates to the nucleus and regulates the transcription of pro- and antiinflammatory cytokines genes, including *Tnfa*, *Il1b*, and *Il6* (56). To ensure global viral spread, we performed intracerebroventricular injections of this viral vector, or an identical control vector expressing eGFP instead of JAK2ca at P0 and waited until the mice were 2 months old before administering i.p. LPS for 2 days at ZT13. Mice treated with AAV/GFAP/JAK2ca showed increased hippocampal IBA1 and GFAP in response to LPS as compared with AAV/GFAP/eGFP-treated animals, demonstrating that activating astrocyte JAK2/STAT3 prior to LPS exposure enhances both micro- and astrogliosis in the brain (Supplemental Figure 5A). However, we observed no increase in BBB permeability at baseline or in response to LPS in the AAV/GFAP/JAK2ca animals (Supplemental Figure 5B). This finding suggests that, while astrocytes respond to LPS independently of microglia, astrocyte activation alone is insufficient to induce BBB breakdown at ZT13 — at least through the JAK2/STAT3 pathway.

## Discussion

While inflammatory BBB breakdown and circadian rhythms in barrier properties have both been explored independently, there is relatively little known about daily rhythms in neuroinflammation and BBB breakdown. Our current findings suggest that there are diurnal changes in the susceptibility of the BBB to inflammatory breakdown, beyond the known rhythms in transporter activity (17, 22–24). Ultrastructural imaging found increased nighttime vasodilation, membrane dysregulation, and vesicle formation — potentially contributing to increased transcytosis and small molecule leak. Transcriptomic analysis showed that these diurnal variations were coupled with dysregulated extracellular matrix–, neuroinflammatory–, and BBB-associated processes, with greater inflammatory gene expression in mice treated with LPS in the evening (ZT13). Persisting glial activation in response to evening LPS was associated with increased BBB permeability, which could be abrogated by microglial ablation but cannot be elicited through astrocyte activation. Thus, the TOD of a systemic inflammatory insult can influence the neuroinflammatory response and BBB integrity.

Since LPS induces BBB breakdown both when administered systemically as well as directly to the CNS, it is difficult to disentangle the role of rhythms in peripheral and central inflammation (33, 57). Brain endothelial cells (BECs) are TLR4^+^, and trace amounts of LPS has been found in them up to 30 minutes after peripheral injection; however, LPS was not found directly in the brain parenchyma (37). This indicates that rather than interacting directly with CNS-resident cells, LPS most likely acts indirectly on peripheral TLR4^+^ cells — including leukocytes as well as BECs — producing cytokines that can readily cross the BBB. Additionally, existing circadian sequencing databases have found that there are no circadian rhythms in *Tlr4* expression in BECs — suggesting that rhythmic neuroinflammatory responses to LPS occurs through CNS-resident cells (21). Further supporting this claim, we found no diurnal variation in peripheral cytokines in our 2-hit LPS model and observed that evening peripheral inflammation remains unaltered in PLX-treated mice (Supplemental Figure 4A), despite the complete ablation of NaFL leak (Figure 6E). Additionally, astrocyte activation in the absence of TLR4^+^ microglia indicates that astrocytes are responding to peripherally derived cytokines rather than parenchymal LPS.

Our data show that glia respond differently to LPS in the evening than in the morning, as both astrocytes and microglia showed increased reactivity 24 hours after evening LPS exposure. While microglia are not physically coupled to the NVU, they are still an intimate component of BBB function and increase perivascular localization during disease (Figure 6A) (16). Using the CSFR1 antagonist PLX, we found that microglia are required for evening LPS-induced BBB breakdown, despite persisting astrogliosis. While PLX can exert off-target effects, including inhibition of Flt and C-kit, our observation that PLX did not change LPS-induced inflammation in peripheral organs, but did affect the brain, strongly implicates microglia (58). It is still possible other CNS-resident CSFR1^+^ cells, such as perivascular macrophages (PVMs), play a role in LPS-induced inflammation. During many inflammatory diseases, PVMs increase in number and vessel coverage; however, it is likely they are actually promoting vessel integrity, since their ablation increases leukocyte extravasation (59, 60). Additionally, astrocyte activation in the absence of TLR4^+^ microglia indicates that astrocytes are responding to peripherally derived cytokines rather than parenchymal LPS — further supporting our peripheral cytokine data in Figure 1.

Our studies have several limitations. First, since only 2 TODs were used, we do not know what happens in midday or midnight and cannot comment on the full circadian dynamics of the system. Second, the use of LPS as a model inflammogen may not simulate the events that occur in more complex situations such as bacterial sepsis. While we have attempted to examine diurnal variation in peripheral inflammation, our efforts were not exhaustive, and it remains possible (if not likely) that some blood-borne factor or cell population may be induced more readily at ZT13 in the periphery, and that this factor might mediate the effects we observe in the brain. Future studies employing single-cell RNA-Seq and proteomics of brain and blood following LPS at different TODs are needed to more fully elucidate these underlying mechanisms. Our observations provide an initial description and provide potential cellular mechanisms that may guide future research.

Our findings have several implications for human health. First, they might prompt investigation into diurnal variation in human BBB integrity in the setting of neuroinflammatory diseases. Second, the pathways that mediate this diurnal variation might be targeted therapeutically to prevent BBB breakdown in the setting of inflammatory diseases, particularly at times of peak vulnerability. Finally, diurnal susceptibility to inflammatory BBB permeability might be leveraged to allow better penetration of certain therapeutics, such as antibodies, into the brain. Chronotherapeutic regimens that incorporate information about diurnal BBB permeability dynamics may improve BBB penetration of antiamyloid drugs, chemotherapies, and other agents. Our work may open the door to such technologies.

## Methods

### Sex as a biological variable.

This study examined LPS-induced inflammation, for which there is a known sex effect (61). However, we found no sex effect on LPS-induced BBB breakdown in preliminary experiments; therefore, we used exclusively male mice to reduce variability in the phenotype.

### BBB assays.

In total, 100 μL of 100 mg/mL NaFL (Sigma-Aldrich: F6377) diluted in sterile PBS was injected s.c. Forty-five minutes later, mice were deeply anesthetized with i.p. pentobarbital (150 mg/kg). Blood (100 μL) was collected by cardiac puncture before it was perfused with ice-cold PBS containing 3 g/L heparin. Both hemispheres were dissected into regions and stored in preweighed tubes filled with 500 μL PBS on ice. Small pieces of peripheral and brain tissues were removed, flash frozen, and stored at –80°C for RNA analysis as described below. Tissue was weighed and then homogenized for 3 minutes in a bullet blender, and serum was added to a serum separator tube (BD Microtainer, 365967) before being spun down and diluted 1:200. Tissue supernatant and serum were incubated 1:1 in 2% Trichloroacetic acid (Millipore Sigma, T9159) overnight (4°C). The samples were spun down again and diluted 1:1 in Borate Buffer (Honeywell, 33650) before being read on a plate reader along with a standard curve. Fluorescence per gram of CNS tissue was compared with fluorescence per μL of serum to determine the absolute amount of NaFL that crossed from the periphery to the brain for each mouse.

For other tracer studies, 4 μL/g body weight of 2% EB (Sigma-Aldrich, E2129) was injected retroorbitally; 15 minutes later, the mice were deeply anesthetized and perfused as described above. For biotin studies, mice were anesthetized (as above) and then underwent transcardiac perfusion with 10 mL of PBS + heparin containing 0.5 mg/mL Biotin. They were then were perfused with another 10 mL of 4% paraformaldehyde (PFA, without the tracer). Whole brains were dissected out, stained with labeled streptavidin, and imaged.

### Circadian rhythm disruption models.

For D:D experiments, mice were kept in standard 12:12 light/dark. The day before experiments began, the lights turned off at 6 p.m. and were kept off for the remainder of the experiment (3 days). For all experiments when mice were injected during the dark phase, red light was used and they were kept in darkness until fully anesthetized. Actigraphy was recorded using passive infrared (PIR) wireless sensors (Actimetrics), and circadian analysis was done using ClockLab Analysis software, version 6.1.02.

### Drug administration.

LPS from *E. coli* O55:B5 (Sigma-Aldrich, L6529) was diluted to 2 mg/mL in PBS and stored at –80°C. Immediately before each injection, LPS stock was thawed and diluted to 0.5 mg/mL in PBS before being injected (i.p.) at a dose of 2 mg/kg. Each LPS stock was not thawed more than twice before being discarded. To avoid batch effects, enough LPS was purchased at each time for an average of 3 experimental cohorts to be treated from the same stock. Mice were weighed each day prior to injection, and the weight at the first day was used to determine dosing for the entire experiment.

### IHC and imaging.

IHC antibodies used in this study include: IBA1 (rabbit, Wako, 019-19741, 1:1,000), GFAP conjugated to Alexa Fluor 647 (mouse, Cell Signaling Technologies, 3657S, 1:800), CD31 (rat, BD Pharmingen, 5500274, 1:250), and CLDN5 (mouse, Invitrogen, 35-2500, 1:100).

Mice were anesthetized and perfused as described above. For IHC experiments, 1 hemisphere was dissected into regions, flash frozen, and stored at –80°C for RNA analysis as described below. The other hemisphere was postfixed in 4% PFA for 12 hours (4°C) before being cryoprotected with 30% sucrose in PBS (4°C) for 24 hours. Brains were then sectioned on a freezing sliding microtome in 50 μm serial coronal sections and stored in cryoprotectant solution (30% ethylene glycol, 15% sucrose, 15% phosphate buffer in ddH_2_0). Sections were washed in TBS × 3, blocked for 60 minutes in TBSX (TBS+ 0.4% Triton X-100; Sigma-Aldrich) containing 3% donkey serum, and incubated overnight at 4°C in primary antibodies diluted in TBSX containing 1% donkey serum (Sigma-Aldrich). Sections were then incubated for 1 hour at room temperature in TBSX with 1:1,000 donkey fluorescent secondary antibody and mounted on slides using Fluoromount-G (Southern Biotech, 0100-01) before coverslipping. For all analyses of TJ and endothelial markers, mice were perfused with 4% PFA for 3 minutes, and the whole brain was collected and postfixed in 4% PFA for 4 hours (4°C) before moving to 30% sucrose for 24 hours (4°C). Brains were sectioned and stored as described above. Sections were blocked overnight in 1% BSA, 0.75% Triton X-100, 5% donkey serum in PBS (4°C) before incubating in primary antibody diluted in PBS containing 0.5% BSA, 0.25% Triton X-100 (referred to as PS/2) for 2 days (4°C). Sections were washed 3x in PS/2 before being incubated for 2 hours at room temperature in PS/2 with 1:1,000 donkey fluorescent secondary antibody, washed 2× in PS/2, and mounted as described above.

All fluorescene imaging was done on a Keyence BZ-X810 microscope. In general, laser intensity and exposure times were selected for each cohort of samples after a survey of the tissue, in order to select appropriate parameters that could then be held constant for all slides in that imaging session. These values varied by antibody, but all sections in a given cohort were imaged under identical conditions at the same magnification. For standard image analysis of epifluorescence images (such as determination of percentage of area for antibodies such as anti-GFAP), TIFF image files were opened using ImageJ (NIH) and converted to 8-bit grayscale files. Images with the dimmest and brightest intensity of staining, as well as some midrange examples, were used to determine an appropriate threshold value that could optimally capture the intended staining across all conditions in that cohort, based on the judgment of the investigator. That threshold was then held constant across all images in the cohort, and black and white images of selected regions of interest were generated and quantified as percentage of area stained using the Analyze Particles function. At least 2 adjacent sections per mouse per region were analyzed and averaged.

Perivascular gliosis analysis was done by taking 60× confocal images on a Zeiss LSM700 of blood vessels; the images were then exported and further analyzed using Imaris 10.0. In short, treatment groups were blinded to the investigator, and 3D volumes for each channel were determined per image to account for LPS effects on glial cell morphology, before an extended surface a distance of 8 μm away from blood vessels was applied. The volume of the channel colocalized to the extended surface compared with total channel volume, as well as the volume of the 8 μm extended surface, were measured. Images (40×) were taken with confocal resolution imaging, and 2D maximum intensity projects were used for representative images.

### Plexidartinib.

PLX was purchased from MedChemExpress (Hy-16749) and shipped to Research Diets Inc. PLX was formulated in AIN-76 diet at 400 ppm and irradiated. PLX or control (AIN-76) chow were fed to mice ad libitum for 2 weeks prior to all PLX experiments.

### RNA quantification.

Tissue was homogenized in 500 μL of Trizol, with beads added before running, in a bullet blender for 3 minutes. TRIzol samples were then subjected to chloroform extraction (1:6 chloroform/TRIzol), followed by thorough mixing, and centrifugation at (12,500*g* for 15 minutes). RNA was then extracted from the aqueous layer using the PureLink RNA Mini Kit according to manufacturer’s instructions. RNA concentration was measured on a Nanodrop spectrophotometer; then, cDNA was made using a high-capacity RNA-cDNA reverse transcription kit (Applied Biosystems) with 250 ng to 1 mg RNA per 20 mL reaction. qPCR was performed with ABI TaqMan primers and ABI PCR Master Mix buffer on ABI StepOnePLus or QuantStudio 12K thermocyclers. TaqMan primers (Invitrogen) were used, and mRNA measurements were normalized to β-actin (Actb) mRNA for analysis. For larger experiments, microfluidic qPCR array measurements were performed by the Washington University Genome Technology Access Center (GTAC) using a Fluidigm Biomark HD system, again using TaqMan primers. RNA-Seq was performed and values were normalized as previously described (62). Volcano plots were made using Enhanced Volcano R package, heatmaps were made using the Pretty heatmap package, and scatter plots were made using ggplot. Overrepresentation analysis dot plots of KEGG pathways were generated using the ClusterProfiler and enrichplot R packages.

### TEM.

Mice were anesthetized as described above and perfused with 10 mL perfusion buffer (0.2 mg/mL xylocaine and 20 units/mL heparin) and then 20 mL fixative solution (2.5% glutaraldehyde, 2% PFA, and 0.15M cacodylate buffer with 2 mM CaCl_2_), all kept at 37°C. Tissue was then postfixed in ice-cold fixative solution overnight at 4°C. The brains were embedded in epoxy resin, and the region of interest were cut into 1 × 1 mm squares, 70 nm thick.

All imaging was done on a JEOL JEM-1400Plus Transmission Electron Microscope at 3,000×–5,000× magnification. Capillaries were identified by morphology (<8 μm in diameter) with a circularity ratio < 2. Treatment groups were blinded to the investigator, and TIFF image files were opened using ImageJ (NIH) and analyzed using the measure tool. The scale for each image was set depending on the magnification used. Diameter was determined as the distance across the vessel at the shortest point, luminal irregularity was the measured length of the plasma membrane divided by the estimated circumference using the elliptical tool, TJ length was the average of each measured TJ per vessel divided by the circumference, and vesicles were only counted if they were actively invaginating from the luminal side.

### Viral vectors.

Jak2ca viral vector was made at the Hope Center Viral Vector Core (at Washington University in St. Louis, MO, USA) using mammalian gene collection cDNA for mouse *Jak2* (ID 16452) gifted by Carole Escartin (CEA, Paris, France). Jak2ca cDNA was then packaged into an AAV9 envelope under expression of the truncated GFAabc1d promoter. A GFP tag was also included as a marker of Jak2ca expression. All viruses were administered via bilateral intracerebroventricular injection in newborn P0 pups as described previously (63). Virus (2 μL) was injected at a concentration of 1.3 × 10^13^ gc/mL.

### Statistics.

In all figures, graphs depict the mean ± SEM, and *n* generally indicates the number of animals, unless otherwise noted in the figure legend. For confocal images, each data point depicts the mean of 2 sections with 6 technical replicates taken per section, and each mouse is considered an *n* of 1. For TEM experiments, each data point depicts the mean for 28–32 technical replicates from 1 mouse, and each of these mean values is considered an *n* of 1. An F test was first performed for datasets with a single dependent variable and 2 groups, to determine if variances were significantly different. If not, 2-tailed unpaired *t* test was performed. For datasets with 2 dependent variables, 2-way ANOVA was performed, and if main effect was significant, Tukey multiple-comparison test was then added for appropriate sets of variables. Outliers were identified using Grubbs test and were excluded. Statistical tests were performed with GraphPad Prism software, version 10.0.2. *P* < 0.05 was considered significant.

### Study approval.

All animal experiments were approved by the Washington University IACUC and were conducted in accordance with AALAC guidelines and under the supervision of the Washington University Department of Comparative Medicine. Seven-week-old male C57BL/6J littermate mice were all obtained from the Jackson Laboratory and allowed to acclimate for 1 week in our facilities before all experiments. For viral injection experiments, E18 timed-pregnant CD1 mice were ordered from Charles River. Mice were housed in a 12-hour light/dark cycle (lights on at 6 a.m. and off at 6 p.m.) and allowed ad libitum access to food and water unless otherwise stated during circadian rhythm disruption models.

### Data availability.

RNA-Seq data are freely available online under GEO submission no. GSE263794. Values for all data points in graphs are reported in the Supporting Data Values file.

## Author contributions

JHL designed research studies, conducted experiments, acquired data, analyzed data, and wrote the manuscript. AP, MWK, and CJN conducted experiments. RD designed research studies. ESM designed research studies, oversaw research, provided reagents, wrote the manuscript, and provided funding.

## Supplementary Material

Supplemental data

Supporting data values

## Version 1 11/08/2024

Electronic publication

## References

[1] Heneka MT (2015). Neuroinflammation in Alzheimer’s disease. Lancet Neurol.

[2] Karch CM, Goate AM (2015). Alzheimer’s disease risk genes and mechanisms of disease pathogenesis. Biol Psychiatry.

[3] Kang R (2020). The dual role of microglia in blood-brain barrier dysfunction after stroke. Curr Neuropharmacol.

[4] Keller M (2009). A circadian clock in macrophages controls inflammatory immune responses. Proc Natl Acad Sci U S A.

[5] Lang V (2021). Susceptibility rhythm to bacterial endotoxin in myeloid clock-knockout mice. Elife.

[6] Geiger SS (2021). Feeding-induced resistance to acute lethal sepsis is dependent on hepatic BMAL1 and FXR signalling. Nat Commun.

[7] Marpegan L (2009). Diurnal variation in endotoxin-induced mortality in mice: correlation with proinflammatory factors. Chronobiol Int.

[8] Nguyen KD (2013). Circadian gene Bmal1 regulates diurnal oscillations of Ly6C(hi) inflammatory monocytes. Science.

[9] Halberg F (1960). Susceptibility rhythm to E. coli endotoxin and bioassay. Proc Soc Exp Biol Med.

[10] Gibbs JE (2012). The nuclear receptor REV-ERBα mediates circadian regulation of innate immunity through selective regulation of inflammatory cytokines. Proc Natl Acad Sci U S A.

[11] Gibbs J (2014). An epithelial circadian clock controls pulmonary inflammation and glucocorticoid action. Nat Med.

[12] Silver AC (2018). Daily oscillations in expression and responsiveness of Toll-like receptors in splenic immune cells. Heliyon.

[13] Persidsky Y (2006). Blood-brain barrier: structural components and function under physiologic and pathologic conditions. J Neuroimmune Pharmacol.

[14] Buckley MW, McGavern DB (2022). Immune dynamics in the CNS and its barriers during homeostasis and disease. Immunol Rev.

[15] Peng X (2021). Blood-brain barrier disruption by lipopolysaccharide and sepsis-associated encephalopathy. Front Cell Infect Microbiol.

[16] Mastorakos P (2021). Temporally distinct myeloid cell responses mediate damage and repair after cerebrovascular injury. Nat Neurosci.

[17] Pulido RS (2020). Neuronal activity regulates blood-brain barrier efflux transport through endothelial circadian genes. Neuron.

[18] Daneman R (2012). The blood-brain barrier in health and disease. Ann Neurol.

[19] Jana A (2022). Increased type I interferon signaling and brain endothelial barrier dysfunction in an experimental model of Alzheimer’s disease. Sci Rep.

[20] Erickson MA (2023). Ultrastructural remodeling of the blood-brain barrier and neurovascular unit by lipopolysaccharide-induced neuroinflammation. Int J Mol Sci.

[21] Zhang SL (2021). A circadian clock regulates efflux by the blood-brain barrier in mice and human cells. Nat Commun.

[22] Zhang SL (2018). A circadian clock in the blood-brain barrier regulates xenobiotic efflux. Cell.

[23] Pan W (2002). Selected contribution: circadian rhythm of tumor necrosis factor-alpha uptake into mouse spinal cord. J Appl Physiol (1985).

[24] Pan W, Kastin AJ (2001). Diurnal variation of leptin entry from blood to brain involving partial saturation of the transport system. Life Sci.

[25] Blacher E (2022). Aging disrupts circadian gene regulation and function in macrophages. Nat Immunol.

[26] Haspel JA (2014). Circadian rhythm reprogramming during lung inflammation. Nat Commun.

[27] Sato S (2017). Circadian reprogramming in the liver identifies metabolic pathways of aging. Cell.

[28] Guan D (2018). Diet-induced circadian enhancer remodeling synchronizes opposing hepatic lipid metabolic processes. Cell.

[29] Saunders NR (2015). Markers for blood-brain barrier integrity: how appropriate is Evans blue in the twenty-first century and what are the alternatives?. Front Neurosci.

[30] Yao L (2018). Evans blue dye: a revisit of its applications in biomedicine. Contrast Media Mol Imaging.

[31] Zhang R (2014). A circadian gene expression atlas in mammals: implications for biology and medicine. Proc Natl Acad Sci U S A.

[32] Nakazato R (2017). Disruption of Bmal1 impairs blood-brain barrier integrity via pericyte dysfunction. J Neurosci.

[33] Banks WA (2015). Lipopolysaccharide-induced blood-brain barrier disruption: roles of cyclooxygenase, oxidative stress, neuroinflammation, and elements of the neurovascular unit. J Neuroinflammation.

[34] Hudson N (2019). Dysregulated claudin-5 cycling in the inner retina causes retinal pigment epithelial cell atrophy. JCI Insight.

[35] Espinal ER (2022). Group B Streptococcus-induced macropinocytosis contributes to bacterial invasion of brain endothelial cells. Pathogens.

[36] Lin XP (2020). Macropinocytosis in different cell types: similarities and differences. Membranes (Basel).

[37] Singh AK, Jiang Y (2004). How does peripheral lipopolysaccharide induce gene expression in the brain of rats?. Toxicology.

[38] Jangula A, Murphy EJ (2013). Lipopolysaccharide-induced blood brain barrier permeability is enhanced by alpha-synuclein expression. Neurosci Lett.

[39] Ciesielska A (2021). TLR4 and CD14 trafficking and its influence on LPS-induced pro-inflammatory signaling. Cell Mol Life Sci.

[40] Juskewitch JE (2012). LPS-induced murine systemic inflammation is driven by parenchymal cell activation and exclusively predicted by early MCP-1 plasma levels. Am J Pathol.

[41] Pulido-Salgado M (2018). RNA-Seq transcriptomic profiling of primary murine microglia treated with LPS or LPS + IFNγ. Sci Rep.

[42] Hasel P (2021). Neuroinflammatory astrocyte subtypes in the mouse brain. Nat Neurosci.

[43] Kang SS (2018). Microglial translational profiling reveals a convergent APOE pathway from aging, amyloid, and tau. J Exp Med.

[44] Kraft P (2010). Deficiency of vasodilator-stimulated phosphoprotein (VASP) increases blood-brain-barrier damage and edema formation after ischemic stroke in mice. PLoS One.

[45] Boje KMK (1996). Inhibition of nitric oxide synthase attenuates blood-brain barrier disruption during experimental meningitis. Brain Res.

[46] Bisht K (2021). Capillary-associated microglia regulate vascular structure and function through PANX1-P2RY12 coupling in mice. Nat Commun.

[47] Elmore MRP (2014). Colony-stimulating factor 1 receptor signaling is necessary for microglia viability, unmasking a microglia progenitor cell in the adult brain. Neuron.

[48] Kokona D (2018). Colony-stimulating factor 1 receptor inhibition prevents disruption of the blood-retina barrier during chronic inflammation. J Neuroinflammation.

[49] Jin WN (2017). Depletion of microglia exacerbates postischemic inflammation and brain injury. J Cereb Blood Flow Metab.

[50] Matsui F (2023). Ablation of microglia does not alter circadian rhythm of locomotor activity. Mol Brain.

[51] Vichaya EG (2020). Microglia depletion fails to abrogate inflammation-induced sickness in mice and rats. J Neuroinflammation.

[52] Hu X (2023). Partial ablation of astrocytes exacerbates cerebral infiltration of monocytes and neuronal loss after brain stab injury in mice. Cell Mol Neurobiol.

[53] Haim LB (2015). The JAK/STAT3 pathway is a common inducer of astrocyte reactivity in Alzheimer’s and Huntington’s diseases. J Neurosci.

[54] Kim H (2022). Reactive astrocytes transduce inflammation in a blood-brain barrier model through a TNF-STAT3 signaling axis and secretion of alpha 1-antichymotrypsin. Nat Commun.

[55] Ceyzériat K (2018). Modulation of astrocyte reactivity improves functional deficits in mouse models of Alzheimer’s disease. Acta Neuropathol Commun.

[56] Minogue AM (2012). LPS-induced release of IL-6 from glia modulates production of IL-1β in a JAK2-dependent manner. J Neuroinflammation.

[57] Mayhan WG (1998). Effect of lipopolysaccharide on the permeability and reactivity of the cerebral microcirculation: role of inducible nitric oxide synthase. Brain Res.

[58] Claeys W (2023). Limitations of PLX3397 as a microglial investigational tool: peripheral and off-target effects dictate the response to inflammation. Front Immunol.

[59] Polfliet MMJ (2001). Meningeal and perivascular macrophages of the central nervous system play a protective role during bacterial meningitis. J Immunol.

[60] Yang T (2019). Brain perivascular macrophages: recent advances and implications in health and diseases. CNS Neurosci Ther.

[61] Chistyakov DV (2018). Sex-mediated differences in LPS induced alterations of TNFα, IL-10 expression, and prostaglandin synthesis in primary astrocytes. Int J Mol Sci.

[62] McKee CA (2022). Astrocytes deficient in circadian clock gene Bmal1 show enhanced activation responses to amyloid-beta pathology without changing plaque burden. Sci Rep.

[63] Kim JY (2014). Intracerebroventricular viral injection of the neonatal mouse brain for persistent and widespread neuronal transduction. J Vis Exp.

