# Supplementary material for: Microglia drive diurnal variation in susceptibility to inflammatory blood-brain barrier breakdown
Source: JCI Insight. 2024 Nov 8;9(21):e180081. doi: 10.1172/jci.insight.180081 (PMC11601573; doi:10.1172/jci.insight.180081)
Supplement: Supplemental data [file jciinsight-9-180081-s278.pdf]

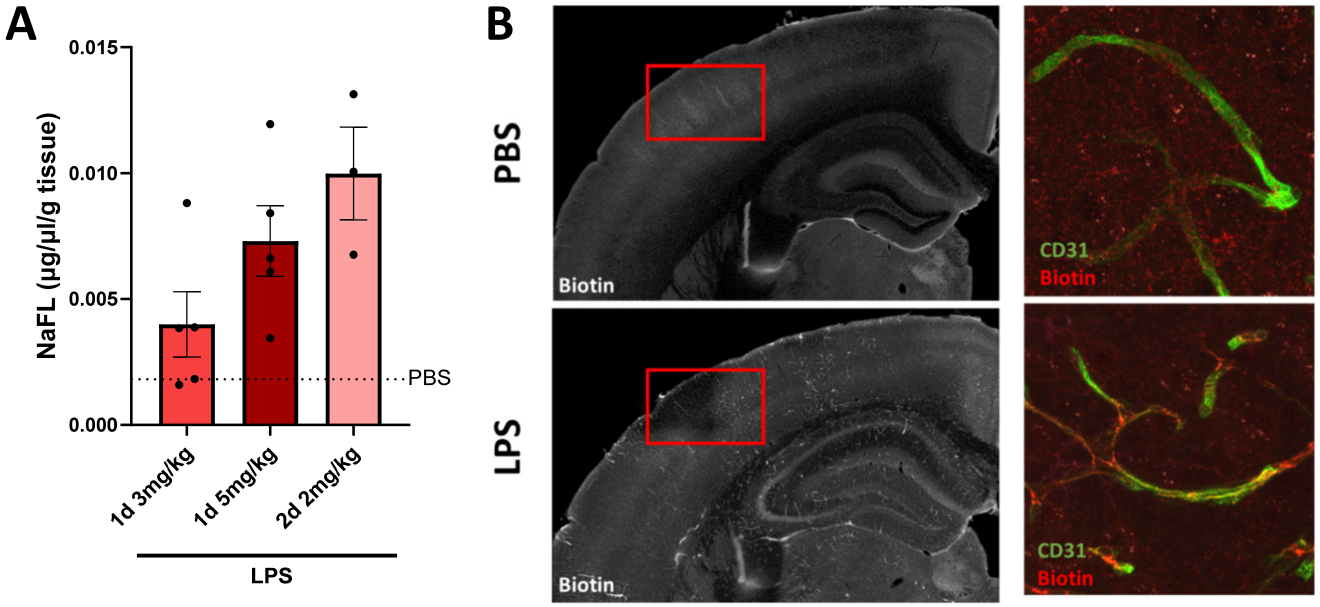

Supplementary Figure 1: Pilot study of LPS effects on BBB permeability to NaFL and biotin. Mice were injected with i.p. LPS at ZT13 on one day or two consecutive days, then injected with NaFL (A) or biotin (B) 24 hours later. In A, NaFL leak into the brain was then quantified. The dotted line indicates the average NaFL leakage after i.p. PBS injection on two consecutive days at ZT13. In B, representative images showing no increased in biotin labeling of blood vessels (CD31) in LPS-treated mice.

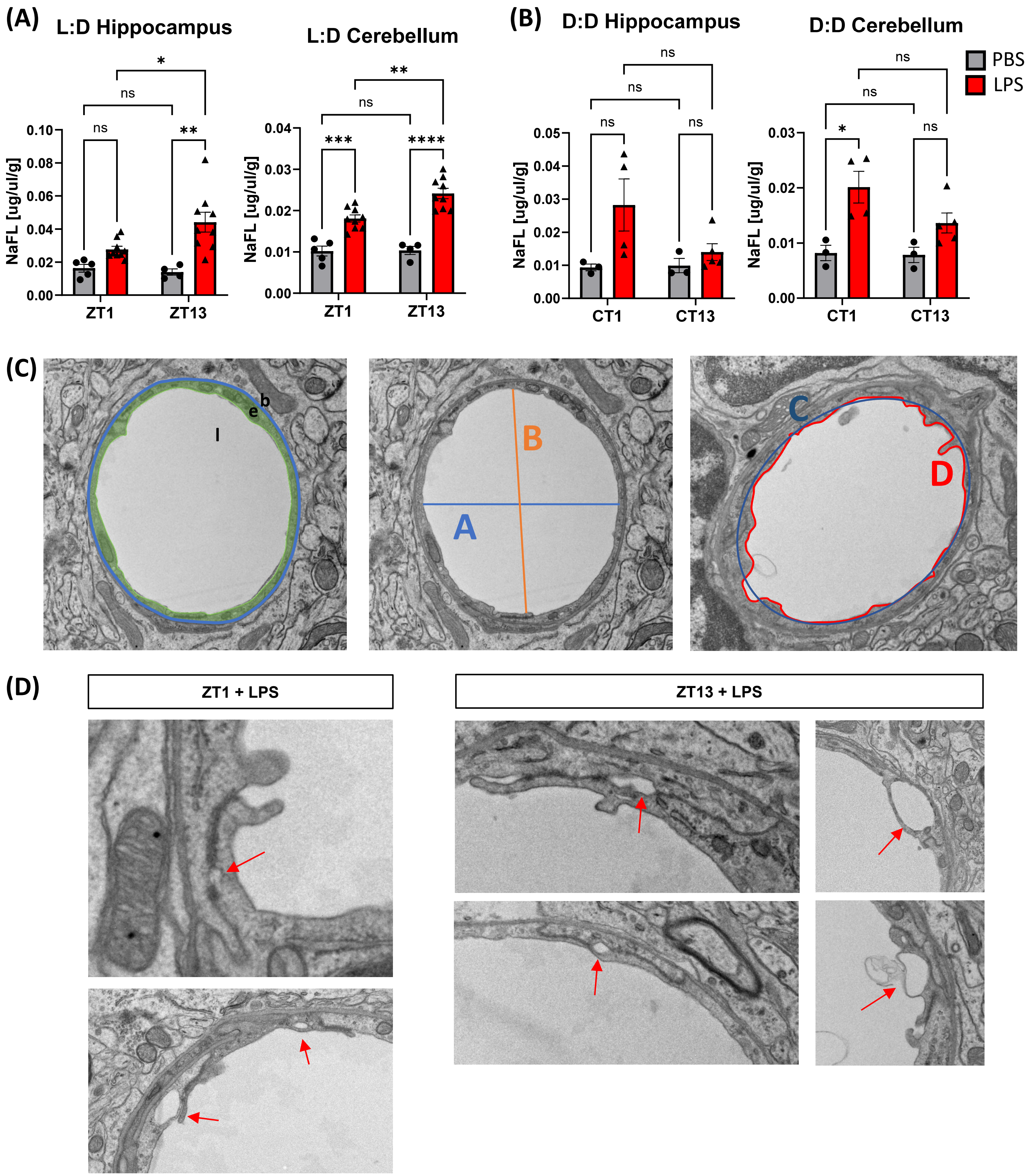

Supplementary Figure 2: TJ disruption in select TEM mice and diurnal variation in BBB leak

(A) TOD differences in NaFL leak in the hippocampus and cerebellum of mice kept in 12h of light and dark (L:D). (B) TOD differences in NaFL leak in the hippocampus and cerebellum of mice kept in constant darkness (D:D) for 1d. n = 3-10 mice per group. (C) Representative images of inclusion criteria for capillaries (b = basement membrane, e = endothelial cell, l = lumen); requirements for shortest diameter (A < 8 um) and vessel circularity (A/B < 2); luminal irregularity measurements (C = elliptical circumference, D = measured circumference). (D) Examples of TJ disruption and vacuole-like structures present in LPS treated mice at both ZT1 and ZT13 denoted by red arrow.

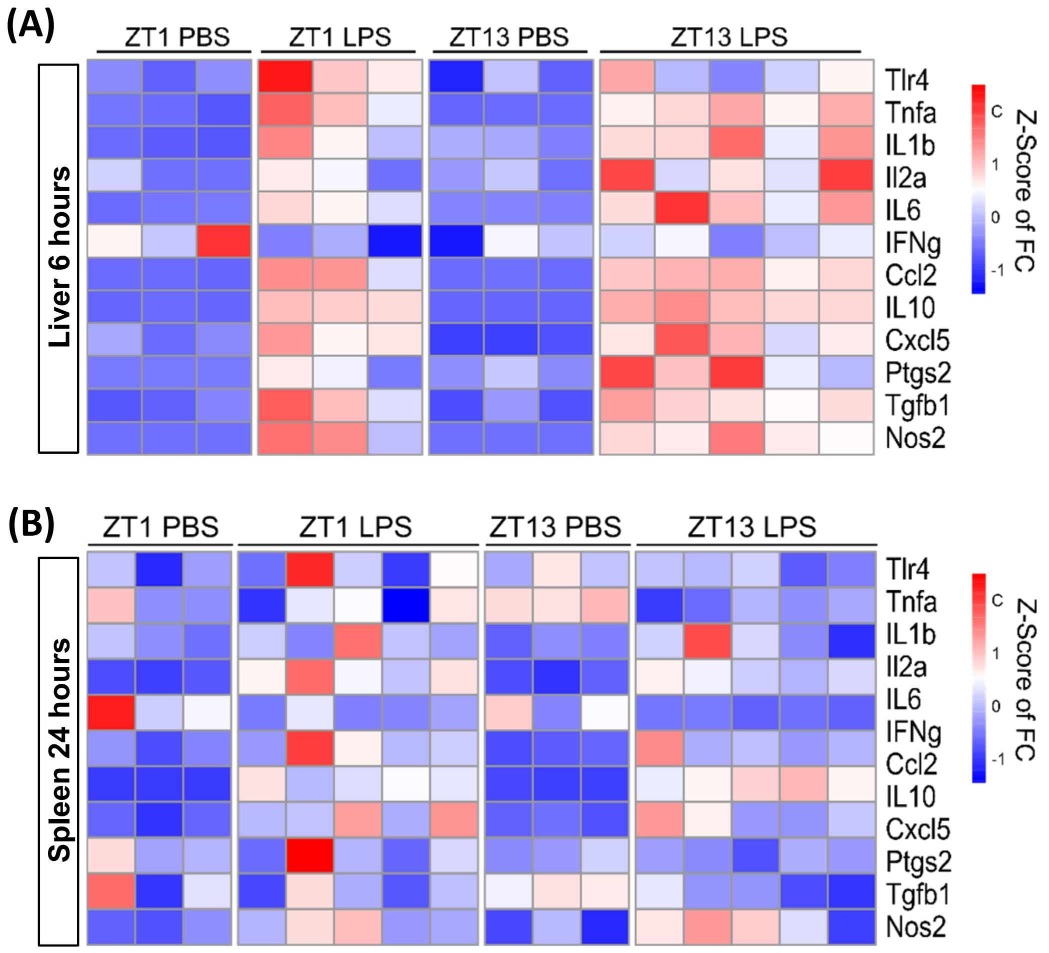

Supplementary Figure 3: Absence of diurnal rhythm in peripheral immune activation at 6 or 24 hours post-LPS. (A) Heatmap of selected gene expression in mouse liver tissue 6 hours after final LPS injection at ZT1 or ZT12. Gene expression is normalized to ZT1 PBS group. (B) Heatmap of selected gene expression in mouse spleen tissue 24 hours after final LPS injection at ZT1 or ZT12. Gene expression is normalized to ZT1 PBS group.

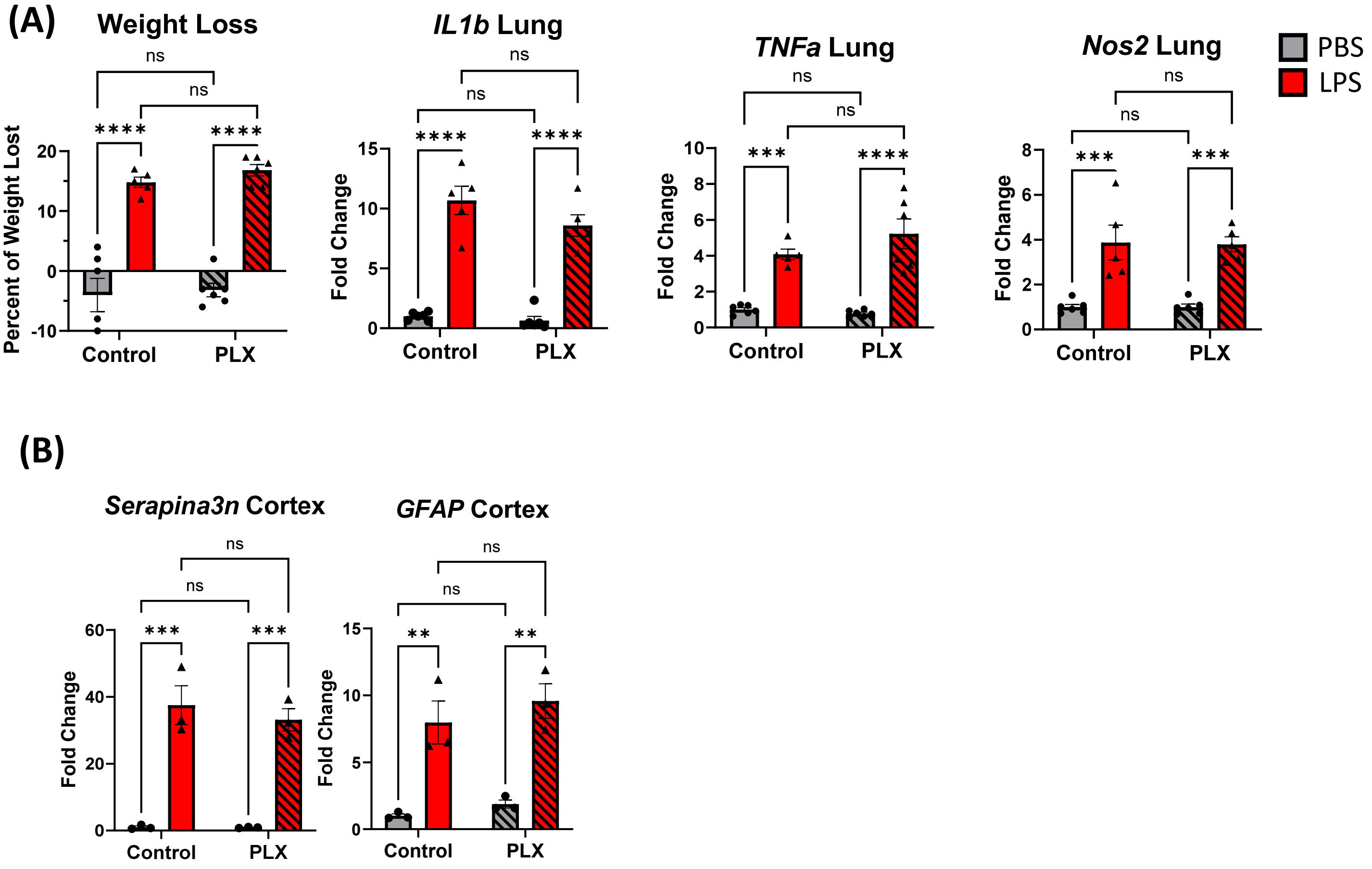

Supplementary Figure 4: PLX does not impact peripheral inflammation or astrocyte gene expression.

(A) Peripheral cytokines and weight loss after 2 weeks of PLX3397 administration followed by 2d of PBS or LPS at ZT13. n = 5-6 mice per group. All graphs have significant main effect of LPS treatment only.

(B) *Serapina3n* and *Gfap* expression in PBS or LPS mice treated with 2 weeks of control or PLX chow. n = 3 mice per group.

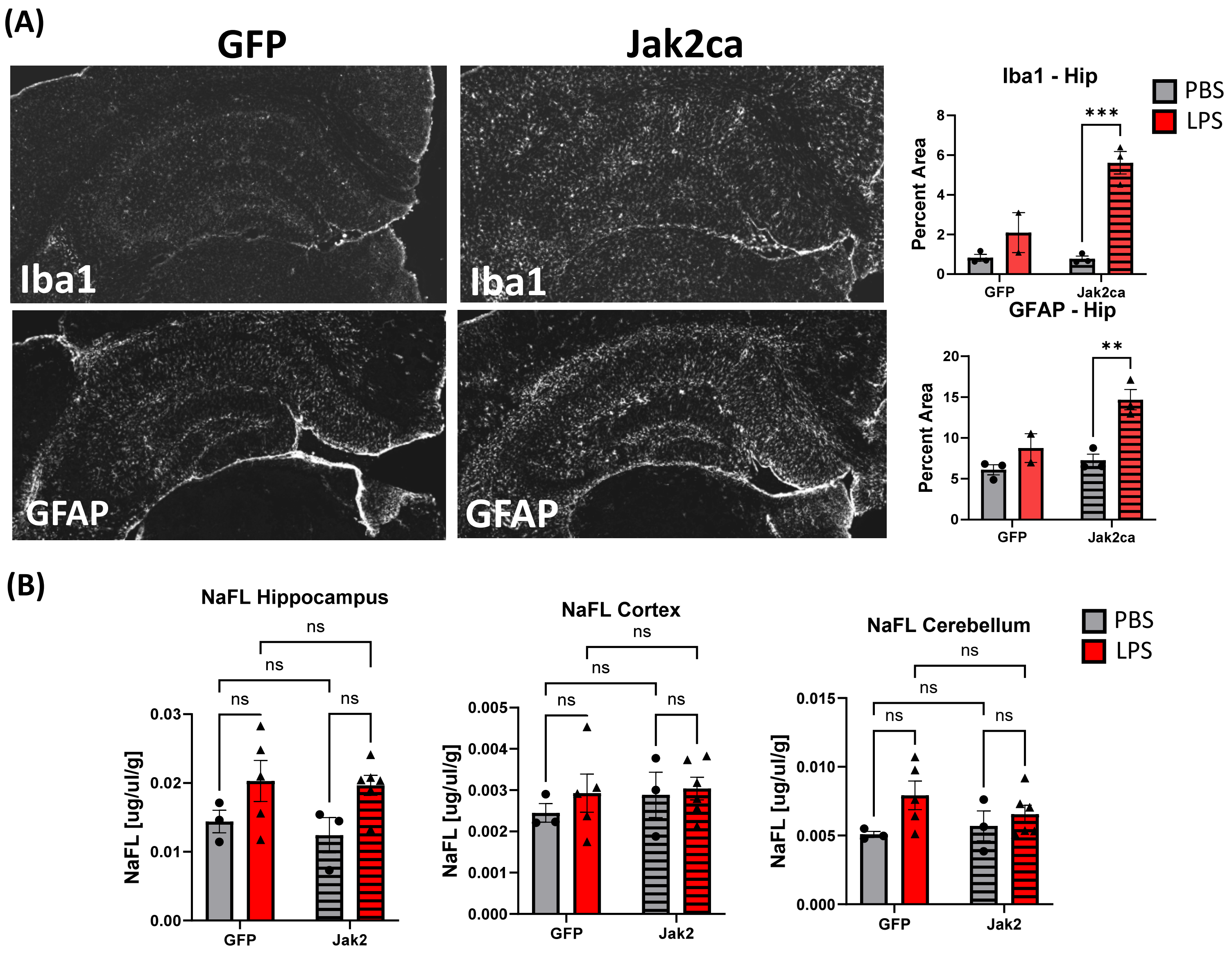

Supplementary Figure 5: Astrocyte activation is insufficient to induce BBB breakdown.
(A) Representative images of IBA1 and GFAP in hippocampus of LPS treated GFP or Jak2ca injected mice, and quantification. n = 2-3 mice per group. (B) NaFL assay in PBS or LPS treated Jak2ca or GFP injected mice. n = 3-5 mice per group.
